# The comparison of insulin resistance frequency in patients with recurrent early pregnancy loss to normal individuals

**DOI:** 10.1186/1756-0500-5-133

**Published:** 2012-03-09

**Authors:** Kotanaie Maryam, Zinatossadat Bouzari, Zahra Basirat, Mehrdad Kashifard, Mahtab Zeinal Zadeh

**Affiliations:** 1 Obstruction &Gynecology Department, Babol University of Medical Science, Babol, Iran; 2 Member of Cellular & Molecular Biology Research Center, Babol University of Medical Science, Babol, Iran; 3 Infertility and Reproductive Health research center, Babol University of Medical Science, Babol, Iran; 4 Department of Internal Medicine, Ayat Allah Rouhani Hospital, Babol University of Medical Sciences, Babol, Iran; 5 Anestesio Department, Babol University of Medical Science, Babol, Iran

## Abstract

**Background:**

Patients with ≥ 3 recurrent spontaneous miscarriages are classified as having RSM. Polycystic ovary syndrome (PCOS) is associated with insulin resistance (IR). The purpose of this study is to evaluate the association of IR and RMS.

**Methods:**

Present case- control prospective study was performed on 100 women in control group (with a history of at a live birth and no history of one more abortion) and study group (with a history of ≥ 3 RMS) who were not diabetes and PCOS. Two groups matched in base of age and body mass index. Blood was withdrawn from the case and control patients for the determination of the fasting blood glucose (FG), fasting insulin (FI) levels and ultrasonography was performed on all the patients.

**Results:**

The observed differences between age, FG and FG to FI ratio levels in case and control groups were not significant (*p *> 0.05) but it was significant about fasting insulin (*p *= 0.0119). FI of < 20 *μu/ml *or ≥ 20 *μu/ml *in case and control group was significant (Chi-square: 4.083, p: 0.0433, odds ratio: 4.4386, CI95% = 1.1541 to 17.0701), whereas the difference between absolute and proportional frequency of patients with FG to FI ratio of < 4.5 and ≥ 4.5 in case and control groups was not significant (Chi-square: 2.374, *p *= 0.123).

**Conclusion:**

Current study showed that in women with RPL, in Iranian race like Americans, frequency of insulin resistance in high, therefore there is a probability of the degree of insulin resistance in women with RPL.

## Background

Recurrent pregnancy loss (RPL) is estimated to occur in 2%-4% of reproductive -age couples [1]. Patients with ≥ 3 recurrent spontaneous miscarriages are classified as having RSM. An RSM remains is a very disturbing event to the affected patients by this health problem; they are always anxious to find the underlying reasons for their miscarriages. This is also a major challenge to the treating physicians [2]. It is a major hazard in pregnancy, both for naturally conceived and those after assisted reproductive technology (ART) treatment [3].

Intensive researches including immunological and genetic studies are still in progress to illustrate the cause of RSM [2]. Chromosome anomaly, uterine malformations or anomalies, hypothyroidism, cervical in competence, anti phospholipid syndrome, bacterial infections and polycystic ovary syndrome (PCOS) are some of the etiological factors associated with RSM [4],[5]. There are some reports of high RSM rates in over weight/obese infertile women treated by ART [6].

Other reports are the condition of PCOS which is probably linked with obesity [7]; this may be due to the high prevalence of overweight/obesity in PCOS women [8]. PCOS is associated with insulin resistance (IR)independent of total or fat- free body mass which can be a key factor behind the link between PCOS/obesity and the risk of spontaneous abortion [3,9]. IR is often increased in 40% women with PCOS [4],[10], and hyperinsulinemia is an etiological factor in the pathogenesis of PCOS [11]. Further studies detected a correlation between increasing insulin resistance and fasting insulin level, with PRL [12]. The exact mechanism of how IR leads to RSM is unknown [2]. IR can be diagnosed by the determination of the fasting glucose to fasting insulin ration, a ratio of < 4.5 being diagnostic of IR [13]. In the present study we were tried to evaluate the association of IR and recurrent pregnancy loss in our area.

## Methods

The present case- control prospective study evaluated between March 2007 and March 2008 in the Department of Obstetrics and Gynecology, Shahid Yahyanejad Hospital, in Iran. At the first 114, patients were evaluated, however, 7women from the study group and 7 women from control group were eliminated due to not recon tact us.

Fifty non pregnant women with ≥ 2 RSM with history, positive serum *B*-HCG and ultrasound documentation of pregnancy, with same sex partner without history of diabetes, were classified as case group. Pregnancy loss was defined as any natural abortion occurring before 24 weeks gestation [1]. Fifty women in reproductive age, non-diabetic, had at least one live birth and/or maximum of one were selected as the control group. The patients of two groups matched in terms of age and body mass index. For the case groups, they completed an evaluation for RSM that included: hysterosalpingogram, serum prolactin, TSH, midluteal serum P, lupus anticoagulant, IgG, IgM, IgA anticardiolipin, antiphosphatidyl serine antibodies, karyotypes on both partners and cervical cultures for Chlamydia, urea plasma, mycoplasma.

Patients with the history of non - consecutive miscarriages, ectopic pregnancy, molar pregnancy, diabetes, multiple partner, PCOS, and current pregnancy were out of the study. The objective and design of this study were explained to all the patients and whole data was completely secret. A written informed consent was obtained from all the participants. The Ethics Committee of the Babol Medical Science University approved the study protocol.

The diagnosis of PCOS patients were based upon the history of having chronic oligoamenorrhea (oligoamenorrhea and amenorrhea were defined as ≤ 6 menses per year and no menses for 1 year retrospectively), clinical and biochemical hyperanderogenism (hirsutism, and severe acne and high levels of total or free testosterone, and rostenedione and DHEAS), abnormal hormonal tests of FSH, LH and those found on ultrasound to contain 12 or more follicles measuring 2 to 9 mm in diameter.

Blood was withdrawn from the case and control patients for the determination of the fasting blood glucose (FG) and fasting insulin (FI) levels. The patients had 12-hour overnight fast before the blood was extracted from the patients between 8:00 and 10:a.m. on the day of the test [14]. Fasting serum insulin was determined by a ECL method (Kit: Gbas, Hitachi 2010, Japan). Glucose concentration was determined by the glucose oxides method(Kit: Pars Azmon, Hitachi 2010, Japan). The insulin resistance is diagnosed by the determination of the fasting glucose to fasting insulin ratio, a ratio of < 4.5 or insulin level of ≥ 20 *μ*unit being diagnostic of insulin resistance[12].

### Statistical analysis

The complete data from the two groups were subjected to statistical analysis using Chi-square for determination of a variable between two groups and t-test for the comparison between two qualities using SPSS package.

## Result

At first, 114 patients were evaluated but 7 women from the case group and 7 women from the control group were eliminated due to not recon tact us. Mean and standard deviation of case group were 28.15 and ± 4.76, respectively (between 19 and 41 years).

Based from the results taken from independent Sample t-test about comparison of the mean of age, fasting blood glucose (FG), fasting insulin (FI) and FG to FI ratio levels in case and control is shown in- Table 1-, the observed differences between age, FG and FG to FI ratio levels in case and control groups were not significant (*p *> 0.05) but it was significant about fasting insulin (*p *= 0.0119).

**Table 1:** Comparison of the mean of age, fasting blood glucose (FG), fasting insulin (FI) and FG to FI ratio levels in case and control groups

Parameter	Groups	Frequency	Mean	SD ± Mean	T	P value
Age	Control	50	28/28	4/77	0/568	0/5715
			
	Case	50	28/02	4/57		
			
FG	Control	50	90/56	10/12	1/810	0/0735
			
	Case	50	94/12	9/34		
			
FI	Control	50	12/23	5/64	2/565	0/0119
			
	Case	50	15/20	5/82		
			
FG to FI ratio	Control	50	8/40	3/26	1/726	0/0876
			
	Case	50	7/26	3/28		

Based from the results taken from Chi-square about the comparison of absolute and comparative frequency of patients with FI of < 20 *μu/ml *or ≥ 20 *μu/ml *in case and control group was significant (Chi-square:4.083, *p*: 0.0433, odds ratio: 4.4386, CI95% = 1.1541 to 17.0701), whereas the difference between absolute and proportional frequency of patients with FG to FI ratio of < 4.5 and ≥ 4.5 in case and control groups was not significant (Chi-square: 2.374, *p *= 0.123). Based from the result taken Chi-square test, the difference between absolute and proportional frequency of patients with IR in case and control groups was significant with (*p*: 0.0248,) (Table 2).

**Table 2:** Comparison of absolute and comparative frequency of patients with insulin resistance (IR) in case and control groups

Groups		IR	Non-IR	T
Control	Absolute frequency	4	46	100
	
	Proportional frequency	%8	%92	%100

Case	Absolute frequency	12	38	100
	
	Proportional frequency	%24	%76	%100

Total	Absolute frequency	16	84	100
	
	Proportional frequency	%16	%84	%100

Chi-square = 5.038 *P *= 0.0248 Odds ratio = 3.6486

CI95% = 1.0855 to 12.2642

The comparison of absolute and proportional frequency of patients with BMI ≥ 27 and patients with BMI < 27 and the difference between absolute and proportional frequency of patients with IR in both groups with *P *= 0.75 was not significant.

## Discussion

Recurrent pregnancy loss (RPL) is estimated to occur in 2-4% of reproductive - age couples [1]. A multitude of etiologic factors have been proposed, including: 3.1% genetic (balanced translocations) 20.2% endocrinologic (elevated levels of serum TSH. hyper prolactinemia, luteal phase defects), 21.6% anatomic (congenital uterine anomalies, Asherman's syndrome, leionyomata, uterinePolyps or septa), 25% immunologic (elevated levels of anticardiolipin or antiphospholipid antibodies, lupus anticoagulant) and 5.8%. microbidogic factors [12,15].

Further studies detected a correlation between increasing insulin resistance and fasting insulin level, with PRL. These findings caused the investigation of IR in women with other causes of PRL.Craig et al in their study showed increased prevalence of insulin resistance in women with a history of at least 2 pregnancy losses [12].

Present study was performed on 100 women incase and control group (with a history of at least a live birth and no history of one more abortion) and the study group (with a history of ≥ 2 RPL) who were not diabetic and PCOS. Mean and standard deviation of age were 28.18 ± 4.67 (at least 19 and maximum 41 years old). A group of 21-25 years old with 28 patients (28%) was the most frequent group. And in the age group of 26-30 years old with 26 patients (26%) also depending upon our study, the average of the age height, weight and BMI between case group and control group did not have significant difference which was not unpredictable based on matching control and case. According to Craig et al results [12], also the frequency of patients with high level of FI (FI ≥ 20 *μu/ml*) in case group was significantly more than the control group (22.45% versus 6.21%, odds Ratio 4.4386, CI: 95% = 1.1541 to 17.0701) and frequency of patients with IR is significantly higher than the frequency of these patients in control group (22.49% versus 8.16%, odds Ratio 3.6486, CI 95% = 1.0855 to 12.2642), but no significant difference was detected between the patients with proportion of FG to FI ratio(G:I) less than 4.5 between case and control group. These findings were in harmony with Craig et al. findings. In Crag et al., study's FI in case group was significantly more than control group and the proportion of patients with high FI and IR in case group was significantly more than control group [12].

In our study and Craig et al., study, also versus other studies the patients with undefined spontaneous miscarriages were investigated, all the patients with RPL were under survey [16,17]. May be because of this the most patients has more than one reason for miscarriage [12,15-18]. Consequently there is more probability for presenting more than one cause of miscarriage in the patient in which finding of one responsible cause stops finding of other cause. Therefore in raft of studied patients IR was not detected. Craig et al; study's showed in 11 patients of 38 patients with RPL (28.9%) there is one more cause except insulin resistance for RPL [12].

Different concepts of insulin resistance may cause the different results of this study and other studies. In current study we used Craig et al procedure for insulin resistance concept which means fasting insulin of ≥ 20 *μu/ml *and/or of fasting blood sugar to fasting insulin ratio less than 45. Legro etal., concluded fasting G: I (glucose/Insulin) ratio is maybe useful as a screening test for insulin resistance in obese non-Hispanic white PCOS women, with the highest sensitivity and specificity. The exact mechanism of insulin resistance for RPL is not completely clear but there is some explanation about it [19].

It is possible that PCOS women have a hostile uterine environmental milieu which causes decrease conception and/or early miscarriages. Elevated PAI-I (plasminogen activator inhibitor-I, an endogenous inhibitor of fibrinolysis) levels have been independently associated with recurrent miscarriages in PCOS women [20].

Metformin enhances luteal phase uterine vascularity and blood flow and reduce the rate of first trimester spontaneous abortions [21,22]. Metformin therapy throughout pregnancy in women with PCOS reduces the otherwise high rate of first-trimester spontaneous abortion [14]. Some in vitro data demonstrate that insulin and glucose modulate glucose transporter at the cellular level of the placentaltrophoblast. Thus maternal insulin and glycemic status may influence the expression of GLUTI [23], also some researchers showed the rule of PA inhibitor which is elevated in women with RPL [24,25]. Increases by elevation of insulin level and decreases with insulin, sensitizator drugs [14,26]. Maybe elevation of this component changes the trombotic process and causes failure in placenta and at last loss of pregnancy [12].

## Conclusion

Current study showed that in women with RPL, in Iranian race like Americans, frequency of insulin resistance in high, therefore there is a probability of the degree of insulin resistance in women with RPL. We recommend that glucose and fasting insulin levels in serum should be measured in all women with RPL. Also another, study in other similar studies in different population is necessary to confirm these findings and the effect of reluctant plasma glucose concentrations drugs in women with history of RPL which should be evaluated in further studies.

Present study involved in some limitation for example, this study done just in one hospitals in Babol city and doesn't cover all hospitals. We suggest to future researchers to cover big population.

## Competing interests

The authors declare that they have no competing interests.

## Authors' contributions

Each author has participated actively and sufficiently in this study. MK conceived the idea and design of the study, interpretation of data, and drafted the manuscript. ZB conceived the idea and design of the study. ZB and ZB made substantial contribution to analysis and interpretation of data. AN and MM have made contribution to collecting of data and editing. Each author revised critically the manuscript and provided final approval of the version to be published.

## Source of finding

Shahid Yahyanejad Hospital
